# Fabrication of Porous Scaffolds with a Controllable Microstructure and Mechanical Properties by Porogen Fusion Technique

**DOI:** 10.3390/ijms12020890

**Published:** 2011-01-25

**Authors:** Qinggang Tan, Songgang Li, Jie Ren, Chu Chen

**Affiliations:** 1 Institute of Nano- and Bio-Polymeric Materials, School of Materials Science and Engineering, Tongji University, 1239 Siping Road, Shanghai 200092, China; E-Mails: renjie65@163.com (J.R.); yourchenchu@163.com (C.C.); 2 Key Laboratory Advanced Civil Engineering Materials, Ministry of Education, School of Materials Science and Engineering, Tongji University, 1239 Siping Road, Shanghai 200092, China; 3 Department of General Surgery, Xinhua Hospital, School of Medicine, Shanghai Jiaotong University, 1665 Kongjiang Road, Shanghai 200092, China; E-Mail: jxlisg@yahoo.cn

**Keywords:** tissue engineering, composite scaffolds, mechanical property, porogen fusion technique

## Abstract

Macroporous scaffolds with controllable pore structure and mechanical properties were fabricated by a porogen fusion technique. Biodegradable material poly (d, l-lactide) (PDLLA) was used as the scaffold matrix. The effects of porogen size, PDLLA concentration and hydroxyapatite (HA) content on the scaffold morphology, porosity and mechanical properties were investigated. High porosity (90% and above) and highly interconnected structures were easily obtained and the pore size could be adjusted by varying the porogen size. With the increasing porogen size and PDLLA concentration, the porosity of scaffolds decreases, while its mechanical properties increase. The introduction of HA greatly increases the impact on pore structure, mechanical properties and water absorption ability of scaffolds, while it has comparatively little influence on its porosity under low HA contents. These results show that by adjusting processing parameters, scaffolds could afford a controllable pore size, exhibit suitable pore structure and high porosity, as well as good mechanical properties, and may serve as an excellent substrate for bone tissue engineering.

## 1. Introduction

Tissue engineering aims to create medical devices that will repair, restore or regenerate tissues impaired by disease, injury, or age [1]. These devices are typically created by seeding a scaffold with cells. A scaffold has always been a key component in the tissue engineering field as it serves as the substrate for implanted cells and the physical support in forming new tissue [2,3]. Ideally, a tissue engineering scaffold needs to have adequate mechanical stability, mechanical strength and be of a highly open porous structure, in order that tissue can develop and tissue fluids and nutrients can transfer freely; pore size and its distribution should be suitable for cell in-growth [4–6]. The tissue engineering scaffold also needs to be easily fabricated into a variety of shapes and sizes. The materials for tissue engineering scaffolds should have controlled biodegradability or bioresorbability, so that scaffolds will eventually be replaced by the forming new tissue [7]. Therefore, the construction of scaffolds with controlled pore structures and mechanical properties is of crucial importance in the tissue engineering field.

In recent years, several techniques have been developed to fabricate tissue engineering scaffolds, including porogen leaching [8–10], gas expansion [11], emulsion freeze-drying [12], thermally induced phase separation [13–16] and 3D-printing [17], *etc.* Compared to other techniques, the porogen leaching technique provides easy control of pore structure and has been well established in the preparation of porous scaffolds for tissue engineering. This technique involves the casting of a polymer/porogen composite followed by aqueous washing out of the incorporated porogen. The pore size, porosity and pore morphology can be easily controlled by the properties of porogen. However, this technique may cause a problem of residual porogen in scaffolds, which may lead to poor pore interconnectivity and harm the cell seeding and culture. Moreover, only thin scaffold cross-sections can be produced due to the difficulty in removing salt particles deep in the matrix.

To avoid these disadvantages, a modified porogen leaching method was developed [18,19]. Only one procedure has proven viable: before the solvent casting process, the porogen fusion step is carried out under certain circumstances. This is called the porogen fusion technique. By use of this technique, a scaffold with desirable pore size, suitable porosity and pore morphology can easily be fabricated. The pore interconnectivity can also be greatly improved and the porogen residual problem is easily solved.

Biodegradable polyesters, such as poly (lactic acid) (PLA), poly (glycolic acid) (PGA) and their copolymers (PLGA), have been widely used for the preparation of tissue engineering scaffolds due to their good biodegradability and processing properties [20–22]. However, they are over flexible and of insufficient strength to meet the mechanical demands of bone replacement, nor do they present a favorable surface for cell attachment and proliferation because of their lack of specific cell-recognizable signals [23,24].

As one of the primary components of extracellular bone matrix, apatite has high strength and exhibits unique advantages when used in bone reconstruction. Apatite also has good osteoconductivity [25], and high affinity to living cells [26]. Therefore, composites composed of synthetic biodegradable polymers and apatite, combine the bioactivity of bioactive apatite and the biodegradability of the polymer. These advantages cause an increasing interest in developing biodegradable polymer/apatite composites for repair, regeneration and tissue engineering of bone. Of all the composites, PLA or PLGA/calcium phosphate have been most extensively studied [9,14,26–28]. With the addition of calcium phosphate in composites, the mechanical strength is not only improved, but this can also lead to a more stable pH value than that of pure PLA or PLGA, due to the fact that the acidic degradation byproducts of PLA or PLGA are buffered by the calcium phosphate.

In this study, poly (d, l-lactide) (PDLLA) scaffolds with controllable structures were first fabricated by porogen fusion technique, and the effects of porogen size, polymer concentration on the microstructure and properties of scaffolds were investigated. Then, the (PDLLA)/hydroxyapatite (HA) composite scaffolds were prepared and the effect of HA on the structure and properties of composite scaffolds was also studied.

## 2. Results and Discussion

### 2.1. Effect of Porogen Sizes on the Structure and Mechanical Properties of Scaffolds

**Figure 1.**
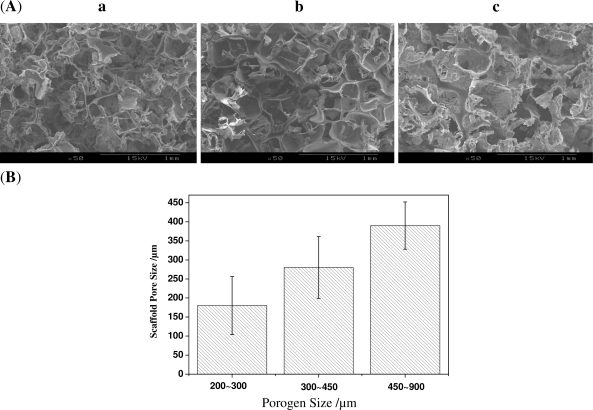
(**A**) Scanning Electron Microscopy (SEM) images of scaffolds with the same Biodegradable material poly (d, l-lactide) (PDLLA) concentration of 15% and different porogen sizes: (**a**) 200∼300 μm (**b**) 300∼450 μm (**c**) 450∼900 μm; (**B**) Scaffold sizes with the same PDLLA concentration of 15% and different porogen sizes calculated from the SEM images.

The effect of different porogen sizes on the structure of scaffolds was first studied by SEM. As is shown in Figure 1A, the microstructure of scaffolds varies with the different sizes of NaCl particles. Scaffolds fabricated using a porogen size of 200∼300 μm, have irregular pore structures with a large number of holes in pore walls. The ones prepared using a porogen size of 300∼450 μm show comparatively regular pore structure, the pore walls become thicker and the number of holes in pore walls seems to slightly decrease. The scaffolds prepared with the largest porogen size of 450∼900 μm display the largest pore size, well-defined pore structure and very thick pore walls, but only very few intermittent holes in the pore walls, which may indicate that the interconnectivity of the scaffolds became poor. The pore size increases with the increasing sizes of the original NaCl particles (Figure 1B). However, the mean pore size is always smaller than that of NaCl particles. For example, the mean scaffold size with the porogen size of 300∼450 μm is only about 180 μm.

The macroporous polymer network was the replica of the surface of the bonded NaCl particles. In other words, the hollow space of the fused NaCl network formed the continuous skeleton of the scaffolds, and the pores were interconnected through the openings formed by the bonding areas of the NaCl. The fusion technique successfully fused porogen together and resulted in the formation of holes in pore walls of scaffolds. However, the same fusion treatment to porogen particles with different sizes may lead to different results. When the porogen size is small, it is easier to moisten the particles and for them to fuse, which makes porogen shape inequable and leads to less hollow space in the fused porogen network. While larger porogen are not so easy to fuse, the shape of the porogen is better retained and the hollow space in the fused porogen networks is comparatively larger. More hollow space means that more polymer solution could be introduced to the scaffolds. On the other hand, when the size of the pore is smaller, the pore wall is subjected to a larger surface tension and this leads to more shrinkage. As a result, under the same fusion conditions, porogen with a smaller size resulted in disorganized pore structure and thinner pore walls; while porogen with a larger size resulted in well-defined pore structure and thicker pore walls. For similar reasons, this resulted in a substantial difference between the pore size and the size of NaCl particles.

**Figure 2.**
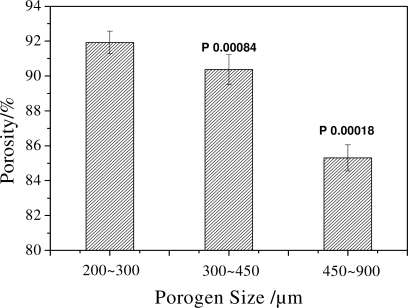
The porosity of scaffolds with the same PDLLA concentration of 15 wt% and different porogen sizes.

The effect of porogen with different sizes on the porosity of scaffolds is shown in Figure 2. It could be seen from the results that the porosity of scaffolds decreases with the increasing size of the porogen (*P* < 0.05). The results are consistent with the SEM analysis. When the size of porogen is small, the stacking density of porogen in a certain space is high compared to that of larger porogen. Moreover the porogen fusion treatment made the density higher; as a result, the space where the porogen was leached out became big. All these factors led to the result that porogen with small size creates a scaffold with high porosity.

**Figure 3.**
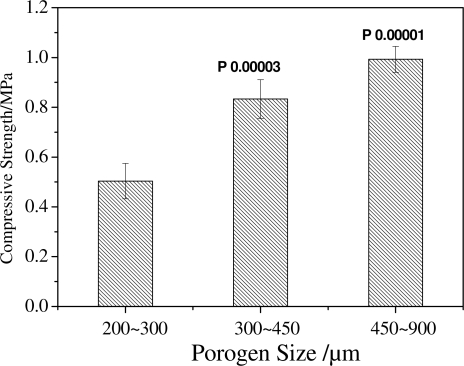
The compressive strength of scaffolds with the same PDLLA concentration of 15 wt% and different porogen sizes.

The compressive strength of scaffolds tends to increase with the increasing size of porogen, as shown in Figure 3. When a porogen size of 200∼300 μm was used, the compressive strength of scaffolds is about 0.5 MPa; but when a porogen size of 450∼900 μm was used, the compressive strength reaches 1 MPa (*P* < 0.01 compared with the size of 200∼300 μm). But the change of compressive strength between 300∼450 μm and 450∼900 μm is not so obvious compared with that of between 200∼300 μm and 300∼450 μm. Scaffolds prepared with porogen of a larger size have thicker pore walls and fewer small pores in the walls, which leads to the result that mechanical properties of scaffolds increase with the increasing size of porogen. When the small pores in the walls decrease, the effect of porogen size on the compressive strength also decreases, which led to the difference of compressive strength between 300∼450 μm and 450∼900 μm decrease. It is clear that the porogen size is a crucial parameter not only for controlling the pore structure, but also for adjusting the porosity and the mechanical properties of scaffolds.

However, these results are different from those found using sugar as porogen [18]. In this literature, using large sugar particles as porogen, the compressive strength, decreased with the increasing size of porogen. The difference in fusing ability and particle size between the salt particle and sugar particle may result in a different flowing behavior of the polymer solution and the pore structure of scaffolds. These factors perhaps led to the constrasting results.

### 2.2. Effect of PDLLA Concentration on the Structure and Mechanical Properties of Scaffolds

**Figure 4.**
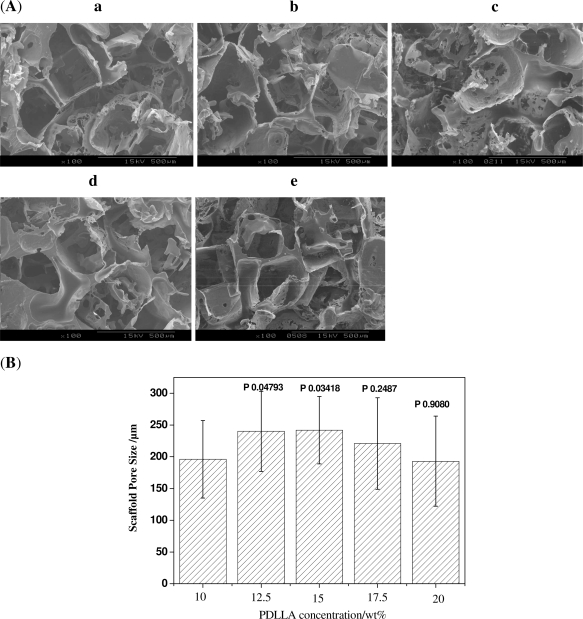
(**A**) SEM images of scaffolds with the same porogen size of 300∼450 μm and varying PDLLA concentrations: (**a**) 10 wt%; (**b**) 12.5 wt%; (**c**) 15 wt%; (**d**) 17.5 wt%; (**e**) 20 wt%; (**B**) Scaffold sizes with the same porogen size of 300∼450 μm and varying PDLLA concentrations calculated from SEM images.

To study the effect of PDLLA concentrations on the properties of scaffolds, a series of scaffolds were prepared with the PDLLA concentration ranging from 10 wt% to 20 wt%. SEM observation (Figure 4A) shows that the pore size of scaffolds prepared from a 10 wt% PDLLA solution is almost the same as that from the 17.5 wt% or 20 wt% PDLLA solution (*P* > 0.05); and the scaffold prepared from 12.5 wt% or 15 wt% has a slightly larger size (*P* < 0.01 compared with that from the 10 wt% PDLLA solution as shown in Figure 4B). However, the scaffolds prepared from the 17.5 wt% or 20 wt% PDLLA solutions have a slightly different morphology from the scaffolds from 10 wt% or 12.5 wt% solutions, *i.e.*, the scaffolds pore walls are thicker.

**Figure 5.**
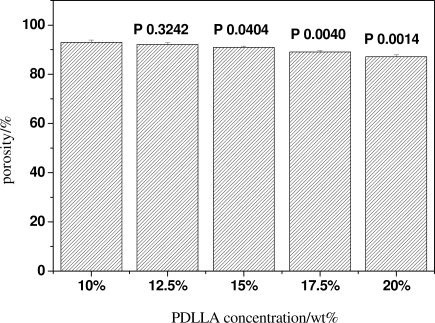
The porosity of scaffolds with the same porogen size 300∼450 μm and different PDLLA concentrations.

**Figure 6.**
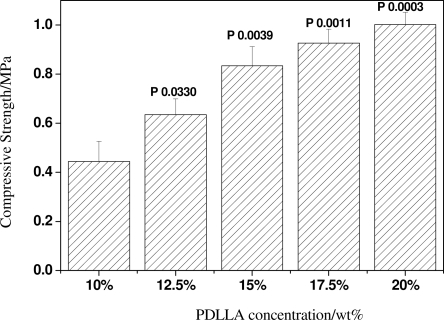
The compressive strength of scaffolds with the same porogen size of 300∼450 μm and different PDLLA concentrations.

The thickness of the pore walls has certain effects on the porosity and mechanical properties of the scaffolds. The porosity and compressive strength test results show the porosity of scaffolds decreases from approximately 93% to 87% with increasing the concentration of PDLLA from 10 wt% to 20 wt%, while its compressive strength exhibits a sharp increase from 0.4 MPa to 1 MPa, as shown in Figure 5 and Figure 6.

The polymer concentration or viscosity has a great effect on the porosity and compressive strength of scaffolds prepared with the fusion salt method [18]. For a defined space, a higher polymer concentration would have more polymers and produce an intact pore wall. These results led to the decreasing porosity and improved mechanical properties of scaffolds. A high polymer concentration also decreases the shrinkage of the pore size during the drying process. As a result, the pore size increased slightly as the PDLLA concentration increased. However, a too high polymer concentration would result in the solution having difficulty flowing into the fused salt scaffold. Consequently, the pore size slightly decreased at high PDLLA concentrations (17.5 wt% or 20 wt%). The decrease in fluidity of the solution at high concentrations had no obvious effect on the porosity and compressive strength of scaffolds prepared from the 17.5 wt% PDLLA solution, compared with that of 20 wt% (the *P* values are 0.0262 and 0.1612, respectively.)

### 2.3. Effect of HA Content on the Structure and Mechanical Properties of Scaffold

Hydroxyapatite (HA) is the principal mineral component of natural bone. It has been extensively used for biomedical implant applications and bone regeneration due to its bioactive, biodegradable and osteoconductive properties. Incorporation of HA into a polymer matrix has often been carried out to increase osteoconductivity and biodegradability with significant enhancement of mechanical strength.

**Figure 7.**
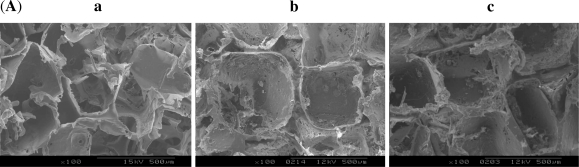

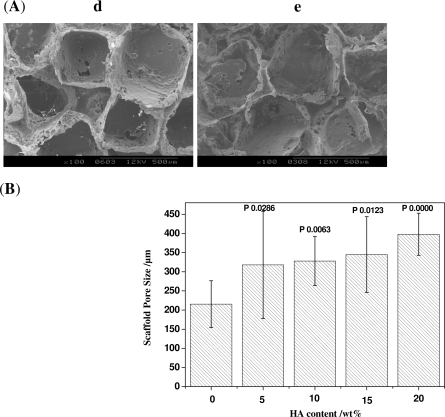
(**A**) SEM images of scaffolds with the same porogen size 300∼450 μm, the same PDLLA concentration 15 wt%, and different HA contents: (**a**) 0 wt%; (**b**) 5 wt%; (**c**) 10 wt%; (**d**) 15 wt% (**e**) 20 wt%. (**B**) Scaffold sizes with the same porogen size 300∼450 μm, same PDLLA concentration 15 wt%, and different HA contents calculated from the SEM images.

The effects of incorporating HA on the structure of scaffolds had also been investigated by varying the HA contents in the PDLLA solutions, while maintaining a constant PDLLA concentration. SEM observations show that the pore structure of scaffolds changes considerably with the varying HA contents (Figure 7). When there is no HA in scaffolds, the pore structure is not so regular and some membrane structures could be observed in scaffolds. But when HA is incorporated, the membrane structure disappears. Moreover, when increasing the HA content, the pore structure of scaffolds becomes more and more regular and the pore walls become thicker. This is presumably due to the high viscosity that HA brought into the PDLLA solution which enhanced the stability of the pore walls. The pore size also appeared to obviously increase when HA was incorporated into the scaffold (All *P* values compared with no HA are less than 0.05). These results have demonstrated that the pore structure of the PDLLA scaffolds can be modified by the incorporation of HA.

**Figure 8.**
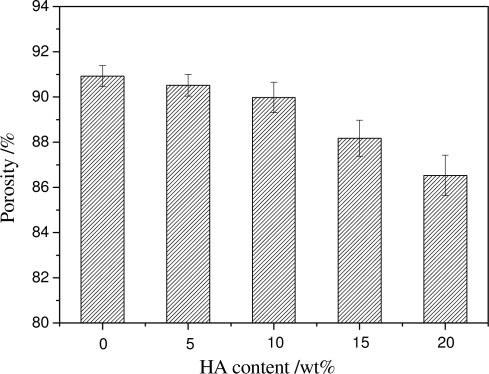
The porosity of the scaffolds with the same porogen size 300∼450 μm, the same PDLLA concentration 15 wt%, and different HA contents.

The content of HA also has some influence on the porosity of the PDLLA/HA composite scaffolds. Figure 8 shows that when the HA content is below 10 wt%, the HA content has little impact on the porosity of the scaffolds, the porosities are all above 90% (All *P* values compared with no HA are larger than 0.05). But when the content of HA is over 10 wt%, the porosity decreases (All *P* values compared with no HA are less than 0.05). Large amounts of HA in the PDLLA solution might cause the agglomeration of the HA particles and block some small interconnected pores, which lowers the pore connectivity and decreases the porosity.

**Figure 9.**
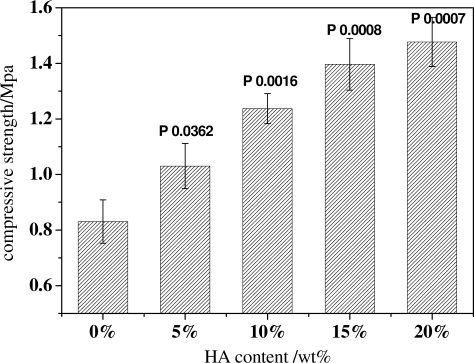
The compressive strength of scaffolds with the same porogen size of 300∼450 μm, same PDLLA concentration of 15 wt%, and different HA contents.

The incorporation of inorganic HA particles in scaffolds can perform as fillers to enhance the mechanical strength of the scaffolds. As seen from Figure 9, the compressive strength of scaffolds increases with the increasing HA content (All *P* values compared with no HA are less than 0.05). With no HA in the scaffolds, the compressive strength of scaffolds was 0.8 MPa. When 5 wt% HA is incorporated, the compressive strength is above 1.0 MPa, which is an increase of over 25%. Even, when the HA content reaches 20 wt%; the compressive strength was near 1.6 MPa, which is double the value of that of the original PDLLA scaffolds. These data demonstrate the positive effects of HA in enhancing the mechanical performance of scaffolds.

**Figure 10.**
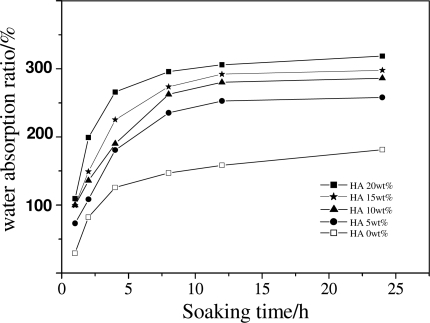
Water absorption ability of PDLLA/HA scaffolds with different HA contents as a function of soaking time.

HA has good hydrophilic properties. Therefore, the introduction of HA in scaffolds would increase the water absorption of the composite scaffolds significantly, as presented in Figure 10. This is probably due to there being a large number of HA particles on the surface of scaffolds and therefore a large fraction of hydroxyl groups could be expected, which leads to an increase in water absorption. Although PDLLA matrix might not be helpful in cell seeding and culture, the incorporation of HA could not only greatly improve the mechanical properties and pore size but also help to enhance the hydrophilicity of the composite scaffolds, which might provide a favorable environment for cell ingrowths.

## 3. Experimental Section

### 3.1. Materials

The PDLLA (Mw = 200,000, PI = 2.3) was synthesized using ring-opening polymerization. The HA nanoparticles, which were acicular crystals of about 100nm in length and 20∼40 nm in width, was prepared by chemical precipitation method [23]. Chloroform and NaCl particles were purchased from the National Chemical Co. (China). The deionized water was twice distilled before use.

### 3.2. Scaffold Preparation

NaCl particles were seized at varying sizes: 200∼300 μm, 300∼450 μm and 450∼900 μm respectively. The molds with diameter 16 mm and height 20 mm were shaped with aluminum foil. The molds were filled with NaCl particles of different size ranges and put in a humidified sealed container, which was placed in an incubator at 37 °C for 10 h. Afterwards the molds together with NaCl particles were dried at room temperature for at least 24 h. Then NaCl particles formed a porous block.

After the formation of the porous block, PDLLA chloroform solution with a concentration of 10∼20 wt% was poured into the molds, allowing the solution to diffuse throughout the mass of porogen. After 2∼3 min, the molds were transferred to a fridge at −20 °C for 2 h to set the mixture. After the solvent was volatilized under room temperature for more than 48 h, the PDLLA was precipitated and NaCl crystals were leached out of the precipitated PDLLA solution in deionized water at room temperature for 3 days. The deionized water was changed approximately 3 times each day. After dried in vacuum for 24 h, PDLLA scaffolds were produced. The PDLLA/HA composite scaffolds were fabricated at the similar way as PDLLA scaffolds. The only difference was that HA was dispersed uniformly in the PDLLA solution before the solvent casting process.

To investigate the effect of the processing parameters on the scaffold properties, each group of scaffolds was prepared by only changing one processing parameter.

### 3.3. Characterization

#### 3.3.1. Morphology

The porous morphology of scaffolds was studied by scanning electron microscopy (SEM, Hitachi S-2360N). The specimens were cut with a razor blade after being frozen in −50 °C conditions for 30 min and then they were coated with gold and observed by SEM at an accelerating voltage of 15 kV.

#### 3.3.2. Porosity

The porosity of scaffolds was measured by liquid displacement method. Absolute ethanol with density ρ was used as a displacement liquid because it can easily penetrate the scaffolds and would not induce shrinking or swelling as a non-solvent of the PDLLA. The specimens, pycnometer and ethanol were kept at 25 °C for 1 h before testing. A pycnometer filled with ethanol was weighed as *W*_1_. A scaffold specimen of weight as *W*_S_ was immersed into the bottle, and then the bottle was submerged slowly until all the air in the scaffold was removed; lastly the pycnometer was refilled with ethanol and weighed as *W*_2_. The scaffold saturated with ethanol was removed from the pycnometer and then the pycnometer was weighed as *W*_3_.

The volume of the scaffold specimen is

(1) $$V_{\text{S}}=(W_{1}-W_{2}+W_{\text{S}})/\rho$$

while the total volume of the pores in it is

(2) $$V_{\text{p}}=(W_{2}-W_{3}-W_{\text{S}})/\rho$$

Then the porosity of the specimen ε is

(3) $$ɛ=V_{\text{p}}/(V_{\text{p}}+V_{\text{S}})=(W_{2}-W_{3}-W_{\text{S}})/(W_{1}-W_{3})$$

#### 3.3.3. Compressive Strength

The compressive strength of scaffolds was evaluated using a computer controlled DXLL-5000 mechanical tester. The top and bottom layers were removed from the original scaffolds to achieve flat surfaces. The testing was carried out according to the national test standards of P. R. China for compression of rigid cellular plastics (GB 8813-1988). Each specimen was made to be 16 mm in diameter and 3 mm thick. The testing was performed under the following conditions: dry/room temperature, a crosshead of 1 mm/min was utilized. The compressive strength of scaffolds is defined as the stress when the specimen is compressed to 30% of its original thickness. Five specimens of each sample were tested.

#### 3.3.4. Water Absorption Ability

The specimens with a dimension of 10 mm × 10 mm × 10 mm were dried and weighed as *W*_1_, then immersed and soaked in twice-distilled water at room temperature (25 °C) for a given time, such as 1 h, 2 h, 4 h, 8 h, 12 h and 24 h. Subsequently, these specimens were taken out of the water; the excess water on the surface of the scaffold was carefully blotted with filter paper and then weighed as W_2_. The water absorption ratio α for specimens at different time is defined as:

(4) $$\alpha =(W_{2}-W_{1})/W_{1}\times 100\%$$

## 4. Conclusions

Biodegradable porous scaffolds were prepared by porogen fusion technique. The effects of porogen size, PDLLA concentration and HA content on the microstructure and properties of the scaffolds were studied. Interconnected open pore structures with small pores in the pore walls are observed. The pore size can be easily controlled by adjusting the porogen size. With the increase of porogen size, PDLLA concentration and HA content, the porosity of scaffolds decreases while the mechanical properties increase. The incorporation of HA greatly improves the pore size, mechanical properties and the hydrophilicity while maintaining the porosity under 10% of HA content in scaffold. The SEM, porosity and compressive strength test results indicate that the microstructure and mechanical properties of scaffolds could be precisely controlled by adjusting the processing parameters.

In conclusion, the microstructure and properties of the scaffolds can be well controlled using porogen fusion technique and the scaffolds may serve as a good substrate in the bone tissue engineering field.

## References

[1] Hutmacher DW (2000). Scaffolds in tissue engineering bone and cartilage. Biomaterials.

[2] Temenoff JS, Mikos AG (2000). Tissue engineering for regeneration of articular cartilage. Biomaterials.

[3] Zhang Y, Zhang M (2001). Synthesis and characterization of macroporous chitosan/calcium phosphate composite scaffolds for tissue engineering. J. Biomed. Mater. Res.

[4] Rizzi SC, Heath DJ, Coombes AGA, Bock N, Textor M, Downes S (2001). Biodegradable polymer/hydroxyapatite composites: Surface analysis and initial attachment of human osteoblasts. J. Biomed. Mater. Res.

[5] Wang M (2003). Developing bioactive composite materials for tissue replacement. Biomaterials.

[6] Karageorgiou V, Kaplan D (2005). Porosity of 3D biomaterial scaffolds and osteogenesis. Biomaterials.

[7] Widmer MS, Puneet K, Gupta PK, Lu L, Meszlenyi RK, Evans GRD, Brandt K, Savel T, Gurlek A, Charles W, Patrick CW, Mikos AG (1998). Manufacture of porous biodegradable polymer conduits by an extrusion process for guided tissue regeneration. Biomaterials.

[8] Mikos AG, Lyman MD, Freed LE, Langer R (1994). Wetting of poly(l-lactic acid) and poly(d,l-lactic-co-glycolic acid) foams for tissue culture. Biomaterials.

[9] Kim SS, Park MS, Jeon O, Choi CY, Kim BS (2006). Poly(lactide-co-glycolide)/hydroxyapatite composite scaffolds for bone tissue engineering. Biomaterials.

[10] Chen G, Ushida T, Tateishi T (2001). Preparation of poly(l-lactic acid) and poly(dl-lactic-co-glycolic acid) foams by use of ice microparticulates. Biomaterials.

[11] Mooney DJ, Baldwin DF, Suh NP, Vacanti JP, Langer R (1996). Novel approach to fabricate porous sponges of poly (dl-lactic-co-glycolic acid) without the use of organic solvents. Biomaterials.

[12] Wang K, Thomas CH, Healy KE, Nuber G (1995). A novel method to fabricate bioabsorbable scaffolds. Polymer.

[13] Nam YS, Park TG (1999). Biodegradable polymeric microcellular foams by modified thermally induced phase separation method. Biomaterials.

[14] Zhang R, Ma PX (1999). Poly(α-hydroxyl acids)/hydroxyapatite porous composites for bone-tissue engineering. I. Preparation and morphology. J. Biomed. Mater. Res.

[15] Hua FJ, Park TG, Lee DS (2003). A facile preparation of highly interconnected macroporous poly (d,l-lactic acid-co-glycolic acid) (PLGA) scaffold by liquid-liquid phase separation of a PLGA-dioxane-water ternary system. Polymer.

[16] Hua FJ, Nam JD, Lee DS (2001). Preparation of a macroporous poly(l-lactide) scaffold by liquid-liquid phase separation of a PLLA/1,4-dioxane/water ternary system in the presence of NaCl. Macromol. Rapid Commun.

[17] Hutmacher DW (2001). Scaffold design and fabrication technologies for engineering tissuesstate of the art and future perspectives. J. Biomater. Sci. Polym. Ed.

[18] Guan L, Davies JE (2004). Preparation and characterization of a highly macroporous biodegradable composite tissue engineering scaffold. J. Biomed. Mater. Res.

[19] Murphy WL, Dennis RG, Kileny JL, Mooney DJ (2002). Salt fusion: an approach to improve pore interconnectivity within tissue engineering scaffolds. Tiss. Eng.

[20] Lu LC, Garcia CA, Mikos AG (1999). *In vitro* degradation of thin poly (dl-lactic-co-glycolic acid) films. J. Biomed. Mater. Res.

[21] Migliaresi C, Fambri L, Cohn D (1994). A study on the *in vitro* degradation of poly (lactic acid). J. Biomed. Sci. Polym. Ed.

[22] Ge Z, Yang F, Goh JCH, Ramakrishna S, Lee EH (2006). Biomaterial and scaffolds for ligament tissue engineering. J. Biomed. Mater. Res.

[23] Barrera DA, Zylstra E, Lansbury PT, Langer R (1993). Synthesis and RGD peptide modification of a new biodegradable copolymer: poly (lactic acid-co-lysine). J. Am. Chem. Soc.

[24] Suh H, Hwang YS, Lee JE, Han CD, Park JC (2001). Behavior of osteoblasts on a type I atelocollagen grafted ozone oxidized poly-l-lactic acid membrane. Biomaterials.

[25] Hench LL (1991). Bioceramics: From concept to clinic. J. Am. Ceram. Soc.

[26] Rizzi SC, Heath DJ, Coombes AGA, Bock N, Textor M, Downes S (2001). Biodegradable polymer/hydroxyapatite composites: Surface analysis and initial attachment of human osteoblasts. J. Biomed. Mater. Res.

[27] Bleach NC, Nazhat SN, Tanner KE, Kellomaki M, Tormala P (2002). Effect of filler content on mechanical and dynamic mechanical properties of particulate biphasic calcium phosphate-polylactide composites. Biomaterials.

[28] Montse CH, Sergio DV, Hentges E, Bleuet P, Lacroix D, Planell JA (2007). Mechanical and structural characterisation of completely degradable polylactic acid/calcium phosphate glass scaffolds. Biomaterials.

